# Controlled vocabularies and ontologies in proteomics: Overview, principles and practice^☆^

**DOI:** 10.1016/j.bbapap.2013.02.017

**Published:** 2014-01

**Authors:** Gerhard Mayer, Andrew R. Jones, Pierre-Alain Binz, Eric W. Deutsch, Sandra Orchard, Luisa Montecchi-Palazzi, Juan Antonio Vizcaíno, Henning Hermjakob, David Oveillero, Randall Julian, Christian Stephan, Helmut E. Meyer, Martin Eisenacher

**Affiliations:** a Medizinisches Proteom Center (MPC), Ruhr-Universität Bochum, D-44801 Bochum, Germany; b Institute of Integrative Biology, University of Liverpool, Liverpool L69 7ZB, UK; c SIB Swiss Institute of Bioinformatics, Swiss-Prot group, Rue Michel-Servet 1, CH-1211 Geneva 4, Switzerland; d Institute for Systems Biology, 401 Terry Avenue North, Seattle, WA 98109, USA; e EMBL-EBI, Wellcome Trust Genome Campus, Hinxton, Cambridge, CB10 1SD, UK; f Indigo BioSystems, Indianapolis, IN 46240, USA; g Kairos GmbH, Universitätsstraße 136, D-44799 Bochum, Germany

**Keywords:** ANDI-MS, Analytical Data Interchange format for Mass Spectrometry, AniML, Analytical Information Markup Language, API, Application Programming Interface, ASCII, American Standard Code for Information Interchange, ASTM, American Society for Testing and Materials, BTO, BRENDA (BRaunschweig ENzyme DAtabase) Tissue Ontology, ChEBI, Chemical Entities of Biological Interest, CV, Controlled Vocabulary, DL, Description Logic, EBI, European Bioinformatics Institute, HDF5, Hierarchical Data Format, version 5, HUPO-PSI, Human Proteome Organisation-Proteomics Standards Initiative, ICD, International Classification of Diseases, IUPAC, International Union for Pure and Applied Chemistry, JCAMP-DX, Joint Committee on Atomic and Molecular Physical data-Data eXchange format, MALDI, Matrix Assisted Laser Desorption Ionization, MeSH, Medical Subject Headings, MI, Molecular Interaction, MIBBI, Minimal Information for Biological and Biomedical Investigations, MITAB, Molecular Interactions TABular format, MIAPE, Minimum Information About a Proteomics Experiment, MS, Mass Spectrometry, NCBI, National Center for Biotechnology Information, NCBO, National Center for Biomedical Ontology, netCDF, Network Common Data Format, OBI, Ontology for Biomedical Investigations, OBO, Open Biological and Biomedical Ontologies, OLS, Ontology Lookup Service, OWL, Web Ontology Language, PAR, Protein Affinity Reagents, PATO, Phenotype Attribute Trait Ontology, PRIDE, PRoteomics IDEntifications database, RDF(S), Resource Description Framework (Schema), SRM, Selected Reaction Monitoring, TPP, Trans-Proteomic Pipeline, URI, Uniform Resource Identifier, XSLT, eXtensible Stylesheet Language Transformation, YAFMS, Yet Another Format for Mass Spectrometry, Proteomics data standards, Controlled vocabularies, Ontologies in proteomics, Ontology formats, Ontology editors and software, Ontology maintenance

## Abstract

This paper focuses on the use of controlled vocabularies (CVs) and ontologies especially in the area of proteomics, primarily related to the work of the Proteomics Standards Initiative (PSI). It describes the relevant proteomics standard formats and the ontologies used within them. Software and tools for working with these ontology files are also discussed. The article also examines the “mapping files” used to ensure correct controlled vocabulary terms that are placed within PSI standards and the fulfillment of the MIAPE (Minimum Information about a Proteomics Experiment) requirements. This article is part of a Special Issue entitled: Computational Proteomics in the Post-Identification Era. Guest Editors: Martin Eisenacher and Christian Stephan.

## Highlights

► The semantic annotation using ontologies is a prerequisite for the semantic web. ► The HUPO-PSI defined a set of XML-based standard formats for proteomics. ► These standard formats allow the referencing of CV terms defined in obo files. ► The CV terms can be used to enforce MIAPE compliance of the data files. ► The mass spectrometry CV is constantly maintained in a community process.

## 1 Introduction

In science the unique definition of the terms used for describing the subject under inquiry is of prime importance to ensure the reproducibility of the analysis and interpretation of the empirically obtained data. A collection of terms for describing a certain modeling domain is called a controlled vocabulary (CV). Around 1735 Carl von Linné [1] introduced the concept of taxonomies into biology for the unique naming of the taxa of animals and plants. These taxonomies complement the controlled vocabularies by adding a hierarchical ordering for the used terms. Later librarians developed the concept of thesauri, which supplements such a hierarchy of terms by relations for similarity and synonyms between the terms. This means that they added other orthogonal dimensions to the mere subordination relation of a hierarchy, which helped them to improve the indexing of literature. Whereas in taxonomies we have only a tree-like structuring of the used terms, thesauri can be used also to represent the collection of terms in a more network- or graph-like structure [2]. Well-known large thesauri in the biomedical area are for instance MeSH (Medical Subject Headings) [3] and ICD (International Classification of Diseases) [4], which are used in medicine for documentation purposes. It has been announced that the next release of the ICD-11 will also be released in a formal ontology format [5].

Ontologies can be seen as a further step in the attempt to structure the terms used in describing a certain domain of interest. Ontologies are used as a means for knowledge representation by defining the objects and concepts as well as their properties and relations used in a modeling domain. Historically ontologies have a long tradition in philosophy, where they were first introduced by Aristotle (384–322 BC) [6] to describe the study of being. Another root of ontologies goes back to computational linguistics, where they are used to avoid interpretation problems due to synonyms, homonyms, acronyms, case ambiguities and misspellings. With the increasing reliance on computing and software in sciences, the need arose to represent the knowledge contained in thesauri in a formal way so that it can be easily processed and interpreted by a computer. Nowadays ontologies are widely used in the modeling of nearly every scientific field to allow easier computational processing of free text, and for defining a unique vocabulary for use in standard data formats. Therefore formal ontologies, which can be seen as the representation of the information contained in a thesaurus, were developed in a variety of formal ontology representation languages that differ by the degree of their expressiveness. In the ideal case the formal ontology has such a rich and formal logic-based expressiveness that it even enables automated reasoning and logic inference processes to take place on the represented data, which lead to the vision of the semantic web [7,8].

In bioinformatics, ontologies are available for many domain areas. An overview about the different ontologies used in biomedicine and bioinformatics, e.g. to ease data integration, is given in [2,9–11] and by the websites of the OBO (Open Biological and Biomedical Ontologies) Foundry [12] (http://obofoundry.org), NCBO (National Center for Biomedical Ontology) BioPortal [13] website (http://bioportal.bioontology.org) or the OLS (Ontology Lookup Service) at the European Bioinformatics Institute (EBI) [14].

In this article we confine ourselves to ontologies in the area of proteomics and show how they are used in the modern XML-based proteomics standard formats defined by the HUPO-PSI (Human Proteome Organization-Proteomics Standards Initiative) consortium. Then using the example of the mass spectrometry ontology PSI-MS [Mayer et al., in submission] we will describe the maintenance of these ontologies and mention important software, editors and tools for use in ontology engineering.

## 2 Standardized formats and ontologies used in proteomics

Standardized formats are important for several reasons. First, more and more journals require that the data underlying a proteomics study should be made public [15–18] either on the journal website or in a public and free repository for mass spectrometry (MS)-based proteomics data like PRIDE [19] (PRoteomics IDEntifications database) or PeptideAtlas [20], which provide long-term storage of the data. In order to ease the task of data submission the EU-funded consortium project ‘ProteomeXchange’ (http://www.proteomexchange.org) was founded. Its goal is to provide a single point of data submission using the community data standard formats and to promote the data exchange between the main MS proteomics data repositories. Furthermore, the use of a standardized format makes it much easier to develop sophisticated software (converters, viewers and other tools) for analyzing the data, because one has to implement readers and writers only for the standard formats and not for the plethora of available proprietary formats. The use of standard formats also makes it easier to compare data from different sources or reproduce the results of analysis. Collaborative projects and fraud detection are made easier. And, in addition, the use of standard formats makes the reuse of data for analysis with improved methods or for answering new research questions more feasible.

JCAMP-DX [21] (Joint Committee on Atomic and Molecular Physical data-Data eXchange format), an IUPAC (International Union for Pure and Applied Chemistry) ASCII-based format, and ANDI-MS/netCDF [22] (Analytical Data Interchange format for Mass Spectrometry/Network Common Data Format), a format originally developed for chromatography-MS data, are older standardized mass spectrometry formats which were developed before the rise of the proteomics era. They are today mainly used in metabolomics for storing and exchanging MS information of small molecules, although it is in principle possible to store proteomics results in them. These two formats make no use of ontologies. The same is true for AniML (Analytical Information Markup Language) [23], an ASTM (American Society for Testing and Materials) standard for representing analytical data, but it is planned that AniML will incorporate parts of the PSI-MS ontology in the future [Mark Bean, personal communication, 2012].

In contrast, the modern XML-based data formats developed by the HUPO-PSI (like mzML [24–26], mzIdentML [27,28], mzQuantML [29,30], TraML [31], GelML [32], spML [33]), PEFF (PSI Extended Fasta Format [34]) and associated standards such as imzML [35,36] are well suited for storing the large data sets encountered in proteomics and allow the referencing of terms from controlled vocabularies defined in ontology files. Other HUPO-PSI formats are PSI-MI [37] for storing molecular interaction data and PSI-PAR [38], a format for describing Protein Affinity Reagents. mzML [24–26] is designed to store data generated by a mass spectrometry experiment; mzIdentML [27,28] captures the process and results of a protein a peptide identification experiment based on mass spectrometry data; mzQuantML [29,30] represents the results of a mass spectrometry quantitative experiment. TraML [31] is an exchange format for defining the transitions used in selected reaction monitoring (SRM), a technique also for quantitative proteomics analysis [39]. GelML [32] and spML [33] are standard formats for describing protein separation techniques. PEFF [34] is a proposed extension for the protein and nucleotide sequence format FASTA [40].

YAFMS [41] (Yet Another Format for Mass Spectrometry) and mz5 [42] are recently proposed non-XML based standards for the storage and exchange of proteomics data sets, which need less space than the unzipped XML-based standard formats. YAFMS stores the data as ‘Blobs’ (Binary Large Objects) in a relational database whereas mz5 uses HDF5 [43] (Hierarchical Data Format) for storing the data, a format especially developed for the storage of very large data sets in high performance computing. Both formats, YAFMS and mz5, allow the referencing of controlled vocabulary terms.

The imzML [35,36] format for MALDI (Matrix Assisted Laser Desorption Ionization) imaging data uses a compromise between data descriptiveness and memory efficiency by storing the metadata part in an XML (.imzML) file, whereas the spectral data are stored in a separate binary format (.ibd) file. Also mzML makes use of the base64 encoding [44] to store the spectra and chromatograms inside the mzML files. This base64 encoding is a method for representing and compressing data as text by encoding them using a subset of 64 characters from the ASCII character set. mzTab [45] is a proposal for a simplified tab-separated-value standard format which allows the use of spreadsheet programs for easily accessing and reporting proteomics identification and quantification results. It is currently in the HUPO-PSI document process [46], which ensures a critical review of proposed standards before their official release. Another tab-based format is MITAB [47], an extension of the PSI-MI format [37].

There are several possible strategies for accessing data in these standard formats. One is the utilization of a common API (Application Programming Interface) [48]. Another possibility is to use standard-specific APIs, as realized for the XML-based formats developed by the HUPO-PSI working group, which developed several Java libraries for the memory-efficient reading and writing of the information contained in the respective standard formats: jmzML [49], jTraML [50], jmzIdentML [51], jmzReader [52] and jmzQuantML [53]. The mzML format is the successor of the merged formats mzData [54] and mzXML [55]. In addition, the alternative de facto standard formats pepXML [56] and protXML [57], which are used by the TPP (Trans-Proteomic Pipeline) [58] for reporting peptide and protein assignments, are still in use. Since the XML-based files have the disadvantage that they can be very large in size, several format reader implementations make use of a sophisticated XPath [59] based XML indexer implemented in the xxindex Java library developed at the EBI (European Bioinformatics Institute) in order to make the processing of these files possible even on standard PCs [49].

An overview about the mass spectrometry standard formats used in proteomics, their usage of CV terms, and their associated web pages is given in Table 1. A more detailed description of some of the standard formats in proteomics is given by the articles of [60] and [Gonzalez-Galarza et al., this issue].

Whereas these standard formats define only the syntax of representing mass spectrometry data, ontologies support flexible definitions of semantics of the represented data. This additional semantic dimension makes the data not only computer readable, but also interpretable by computers, and is a prerequisite for more sophisticated software tools for analyzing and mining the data. The semantic information is defined independently of the standard formats by using ontologies. This means on the one hand that the semantic information can be easily reused by the various standards and on the other hand that it is in principle possible to change the representation format of the semantics without the need for redefining the standard format itself. Furthermore the controlled vocabulary can be extended independently, i.e. without the need to change the structure of the released standard format.

The most important ontologies that can be used to report proteomics experiments are listed in Table 2. They are used by the XML-based proteomics standards defined by the HUPO PSI working groups [61] and some of them can of course be used in other biological disciplines.

It should be mentioned that Unimod [76] is not an ontology in a strict sense – as no relations are defined and therefore no hierarchy is built – and therefore not supported by the OLS (Open Lookup Service). It contains modifications defined by Mascot [78] and converted by a XSLT (eXtensible Stylesheet Language Transformation) [79] script into the obo format.

## 3 Ontology formats

For the formal representation of ontologies several representation formats exist, which differ in their degree of expressiveness. The most important of these are OWL (Web Ontology Language, version 2) [80], RDF(S) (Resource Description Framework (Schema)) [81], Topic Maps [82], Description Logic (DL) [8,83] and the obo flat file text format.

The obo format is used by the open source editor *OBO*-*Edit* [84], which replaced the older *DAG*-*Edit* editor. The obo format [85,86] is the simplest and currently most widespread used ontology format in bioinformatics. Those who are interested in the obo format can subscribe to the dedicated mailing list [87].

The obo format first lists some header tags containing meta-information like for instance the date, the version and other imported ontologies. After the header a list of type definitions, a list of terms and a list of instances follow. The format can contain three types of stanzas: [Typedef], [Term] and [Instance], where each stanza can be described by a collection of allowed tags for the respective stanza type. So the format distinguishes in total between 4 types of tags: header tags, typedef tags, term tags and instance tags. The obo flat file format specification recommends that the [Term], [Typedef], and [Instance] stanzas should be serialized in alphabetical order on the value of their id tag and also for the specification of the tags inside the stanzas a certain order is recommended [86].

As an example within psi-ms.obo, the definition for ‘ionization energy’ (term *MS:1000219*) is shown below. It defines the term together with an identifier, a short human readable definition of the term's meaning, a synonym and the value type for this term. In addition here two relationships are given: the relationship “is_a” states that the ionization energy is a specialization of an ion attribute and the relationship “has_units” states that the ionization energy has to be given in electron volts. Other relationships used in psi-ms.obo are for instance “part_of” and “has_regexp”. The relation “has_regexp” for instance is used to describe the cleavage sites of restriction enzymes. Most terms are by default used as “flat” enumeration types, i.e. with the meaning only given by their name and description. The ‘xref: value-type’ entry allows stating that terms require a value, in this case of type float. An overview about the possible relationships is given in the OBO Relation Ontology [74,88].

*[Term]*
*id MS:1000219*
*name: ionization energy*
*def: “The minimum energy required to remove an electron from an atom or molecule to produce a positive ion.” [PSI:MS]*
*synonym: “IE” EXACT []*
*xref: value-type:xsd\:float “The allowed value-type for this CV term.”*
*is_a: MS:1000507 ! ion attribute*
*relationship: has_units UO:0000266 ! electronvolt*

The usage of this CV term in a standard format file is shown later in Section 5.

As shown in the next example for a quadrupole ion trap, it is possible to define more than one synonym for a given term, which allows to model cases where many terms are in use for the same meaning, so that redundancy on term level is avoided.

*[Term]*
*id:MS:1000082*
*name: quadrupole ion trap*
*def: “Quadrupole Ion Trap mass analyzer captures the ions in a three dimensional ion trap and then selectively ejects them by varying the RF and DC potentials.” [PSI:MS]*
*synonym: “Paul Ion trap” EXACT []*
*synonym: “QIT” EXACT []*
*synonym: “Quistor” EXACT []*
*is_a: MS:1000264 ! ion trap*

Sometimes a merging, splitting, replacement or deprecation of an ontology term is necessary, e.g. due to upcoming new technologies or instruments or changes in standard formats. Montecchi-Palazzi et al. [89] demand that the old terms must be obsoleted, but they must stay inside the ontology and any new terms replacing them must get a new identifier. This is important for backward compatibility, so that instance files with old identifiers are still valid and contain reasonable content. This marking as obsolete is only necessary, if the meaning of a term changes. In contrast, changes in wording only can be made without marking a term obsolete. An example for an obsolete term is for instance:

*[Term]*
*id: MS:1001849*
*name: sum of MatchedFeature values*
*def: “OBSOLETE Peptide quantification value calculated as sum of MatchedFeature quantification values.” [PSI:PI]*
*comment: This term was made obsolete because the concept MatchedFeature was dropped.*
*is_a: MS:1001805 ! quantification datatype*
*is_obsolete: true*

Here the relation “is_obsolete” was added and set to true, the ‘def:’ tag begins with ‘OBSOLETE:’ and the following definition now contains a hint which term should be used instead. In this example it is mentioned that the concept of a MatchedFeature was dropped, so that there is now no need for using the CV term anymore.

Inside the obo file one can also reference terms defined in other ontologies by using database cross reference (“dbxref”) lists. This way, one cannot only refer to other ontologies, but also to databases or web pages. For instance the example term (*MS:1000219*) for the ‘ionization energy’ shown above contains a “dbxref” list after the “def:” term tag, stating the source where the term was originally defined. In the example it references with *[PSI:MS]* to itself. Analogously the relationship “has_units” refers with the “dbxref” ‘*UO:0000266*’ to the “Unit” ontology [77]. Another example would be the term tag *def: “Enzyme leukocyte elastase (EC 3.4.21.37).” [BRENDA:3.4.21.37]*, which states that the BRENDA ontology is the original source of reference for the enzyme “leukocyte elastase”. A list of allowed “dbxref” terms can be found online at the gene ontology website [90].

Other formal languages for ontology representation like OWL [80], RDF(S) [81] and Description Logic (DL) [8,83] allow much more expressive semantics than the relatively simple obo format and can be used for automatic reasoning procedures and are the basis for building up the semantic web [91–93].

Description Logics [8,83] are decidable parts of first-order predicate logics and differ from one another by their degree of expressivity. This means that they have more expressiveness than propositional logic, but decision problems based on them are more efficiently decidable than the general first-order predicate logic. The complexity [94] of the decision problems depends on the different allowed and not allowed language constructs of the used description logic. RDF [81] is based on XML and describes data based on a graph model consisting of triples of subject, predicate and object. Comparable to XML schema for XML, RDFS describes the allowed structures for RDF documents. OWL resp. OWL 2 build up on the top of RDF(S) and are thus more expressive. OWL 2 defines the three so-called “profiles” OWL 2 EL, QL and RL [95] differing in allowed language constructs determining the level of expressiveness. Ontologies for the OBO Foundry must be either in obo or OWL format and must use the OBO Relation Ontology [74]. From the ontologies mentioned in Table 2 only the OBI ontology is in OWL format, all others are represented in the obo format. It should be mentioned here, that several tools exist to automatically convert obo files into some of these other formats like OWL or RDF [96–99]. Of course, the resulting files cannot contain more information than the simple obo files, but they can be used as a starting point for a semantically more detailed modeling of the ontology information.

## 4 Software and tools for accessing, browsing, creating, editing and manipulating ontology files

Because all the formats OBO, OWL, RDF(S) are text files one can in principle edit them with a normal text editor. However, for working more efficiently with them, some specialized editors exist. In addition to an ASCII editor they have additional useful functions, like for instance visualizing the hierarchy or performing some validity checks before storing a changed version of the ontology file. The most important of these specialized ontology editors are listed in Table 3. A good overview about tools for ontology engineering is given in [100].

**Fig. 1:**
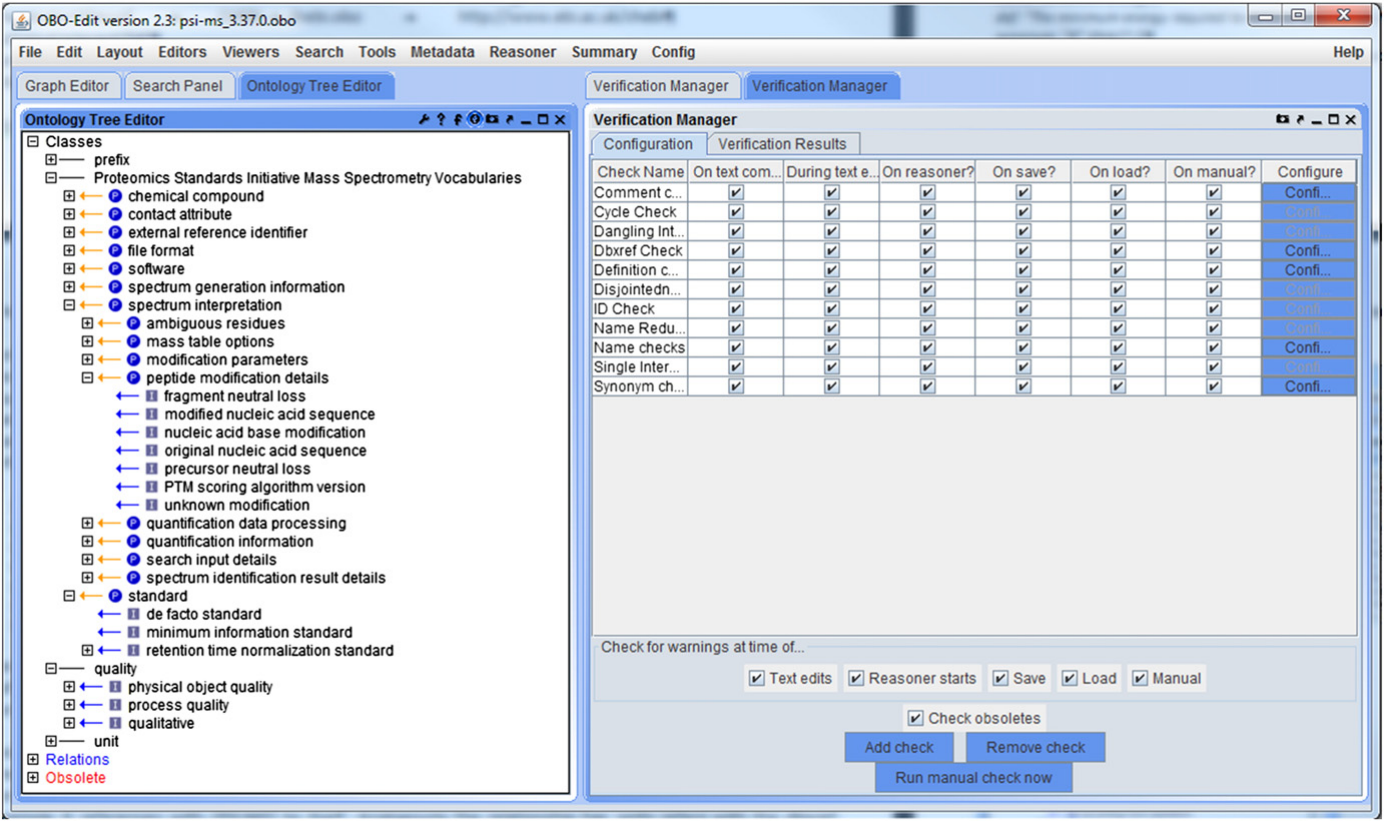
The OBO-Edit [84] user interface showing the ontology tree editor and the verification manager.

*OBO-Edit* [84] for instance contains a configurable verification manager (Fig. 1), where one can specify which checks the editor should perform during loading, saving or changing of an obo ontology file. Whereas *OBO*-*Edit* and OLS [14] work only with files in the obo format, the *Protégé* editor and the *OBO-Explorer* support also OWL. *Protégé* [101] furthermore supports the RDF(S) ontology format. With OLS one can either browse interactively through the ontologies by using the web interface [102] or access them from within a Java class by using the web service implemented in the available ols-client.jar file of the EBI.

For accessing the ontology files, the Open Lookup Service (OLS) [14] allows the browsing, searching and accessing of the obo file contents either interactively via a web-site interface or automatically by computer programs via a web service interface. Internally, OLS uses an indexing based on Apache Lucene [106], for case-insensitive indexing of all the terms and their synonyms [107]. This allows converter programs like PRIDEConverter 2 [108] or ProCon (PROteomics CONversion tool) [109] to easily access the ontology files during the creation process of proteomics data files.

## 5 Use of controlled vocabularies in the XML-based proteomics standard formats of the HUPO-PSI

The HUPO-PSI formats mzML, TraML, mzIdentML, mzQuantML and GelML, as well as the PSI-associated format imzML and the non-XML mzTab [45] and MITAB [47] formats all make intensive use of controlled vocabulary terms defined in ontologies. Therefore these formats allow the usage of < cvParam > elements at various places in an instance data file. All these standard format instance files have at their beginning an element < CvList >, in which the used controlled vocabularies are first defined with their name, their ID, their version and the URI (Uniform Resource Identifier). The latter specifies a name space-like unique identifier and can – if it is a URL – also specify, where to find the actual ontology files:

*< CvList >*
  *< Cv fullName = “Proteomics Standards Initiative Protein Modifications” version = “1.010.7” uri = “**http://psidev.cvs.sourceforge.net/viewvc/psidev/psi/mod/data/PSI-MOD.obo**” id = “MOD”/>*
  *< Cv fullName = “Proteomics Standards Initiative Mass Spectrometry Vocabulary” version = “3.34.0” uri =**http://psidev.cvs.sourceforge.net/viewvc/psidev/psi/psi-ms/mzML/controlledVocabulary/psi-ms.obo* *id = “MS”/>*
  *< Cv fullName = “UNIMOD CV for modifications” version = “1.0” uri =**http://www.unimod.org/obo/unimod.obo*
  *id = “UNIMOD”/>*
  *< Cv fullName = “Unit Ontology” version = “1.0”*
  *uri = “**http://obo.cvs.sourceforge.net/viewvc/obo/obo/ontology/phenotype/unit.obo**” id = “UO”/>*
*</CvList >*

Later in the instance data file these defined controlled vocabularies and their terms can be referenced, as shown in the following mzIdentML example specifying the original result file, the spectra data files, their formats and the used search database using < cvParam > XML elements:

*< Inputs >*
  *< SourceFile location = “D:\TestingProteinGrouping\Testing Decoy-Dash.msf” id = “SF_1”>*
    *< FileFormat >*
      *< cvParam accession = “MS:1001107” cvRef = “MS” name = “data stored in database”/>*
    *</FileFormat >*
  *</SourceFile >*
  *< SourceFile location = “C:\Users\Gerhard\AppData\Local\Temp\Testing Decoy-Dash_2.prot.xml” id = “SF_2”>*
    *< FileFormat >*
      *< cvParam accession = “MS:1001422” cvRef = “MS” name = “protXML file”/>*
    *</FileFormat >*
  *</SourceFile >*
  *< SearchDatabase location = “uniprot_sprot_human_target_decoy.dashed.fasta” name = “uniprot_sprot_human_target_decoy.dashed.fasta” id = “SDB”>*
    *< FileFormat >*
      *< cvParam accession = “MS:1001348” cvRef = “MS” name = “FASTA format”/>*
    *</FileFormat >*
      *< DatabaseName >*
        *< userParam value = “uniprot_sprot_human_target_decoy.dashed.fasta” name = “database name”/>*
      *</DatabaseName >*
  *</SearchDatabase >*
  *< SpectraData location = “D:\HPP_VallHebron_MRMvelos_120719_Fr04_04.mgf” id = “HPP_VallHebron_MRMvelos_Test1_120719_Fr04_04.mgf”>*
  *< ExternalFormatDocumentation>http://www.psidev.info/files/mzIdentML1.1.0.xsd </ExternalFormatDocumentation >*
    *< FileFormat >*
      *< cvParam accession = “MS:1001062” cvRef = “MS” name = “Mascot MGF file”/>*
      *</FileFormat >*
      *< SpectrumIDFormat >*
        *< cvParam accession = “MS:1000774” cvRef = “MS” name = “multiple peak list nativeID format”/>*
      *</SpectrumIDFormat >*
  *</SpectraData >*
*</Inputs >*

**Fig. 2:**
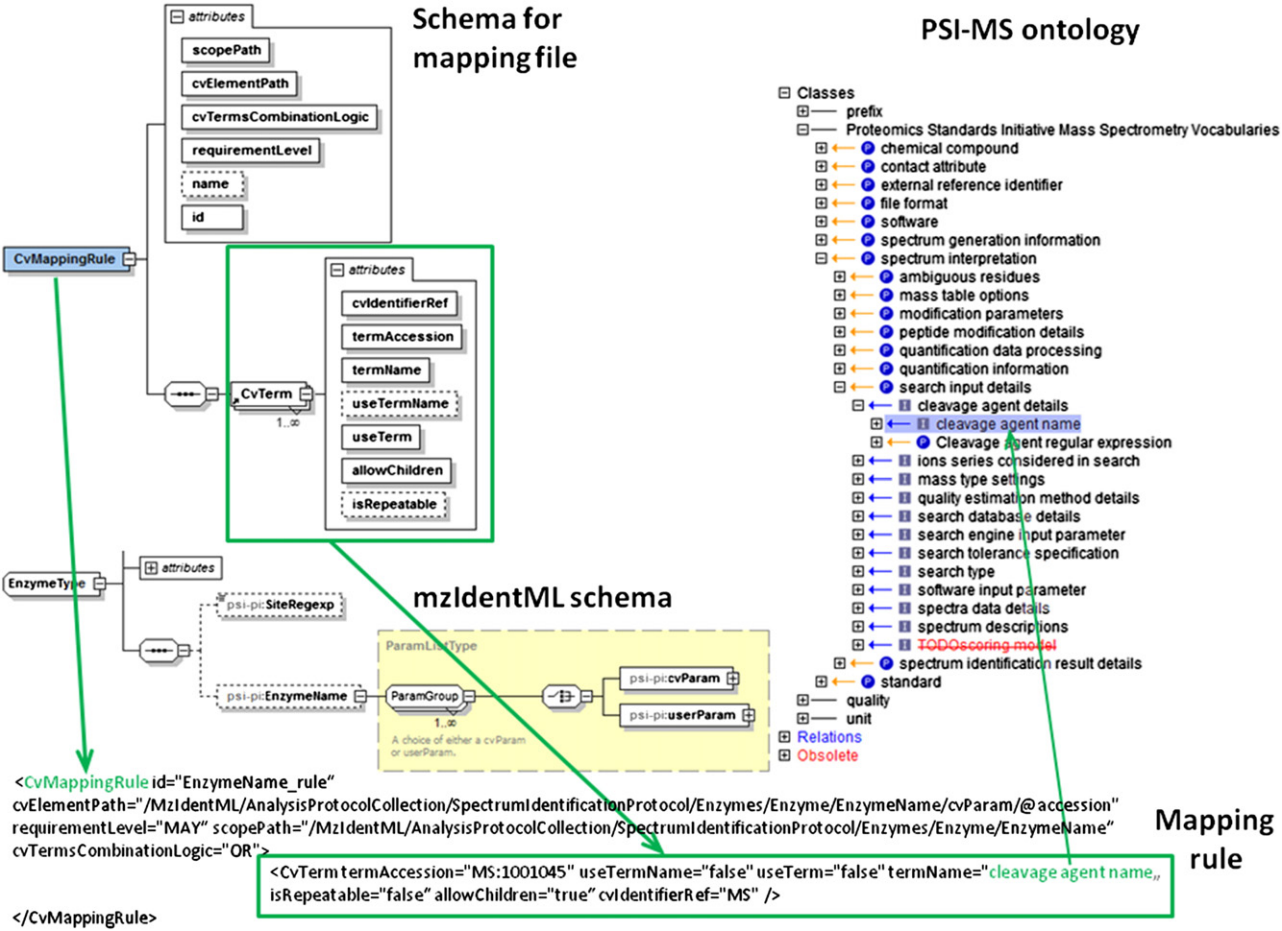
Mapping rule for using a CV term in the correct position (XPath) of an XML data file.

To make sure that the CV terms are used only at correct positions in the files, a mapping file exists for each of the standards, which exactly defines where and in which combination with other CV terms a certain CV term can occur inside the data file. The schema for this CV mapping file is shown in Fig. 2. Such a mapping file contains a < CvReferenceList > element, which contains a list of CVs that are required in an instance data file and a < CvMappingRuleList > element, which contains the mapping rules for the various elements of the data file.

Each < CvMappingRule > element has an attribute ‘cvElementPath’, which describes in XPath expression syntax [59] the path to the element in the standard file to which the current CV mapping rule applies. The attribute ‘cvTermsCombinationLogic’ is a Boolean operator describing how the subordinate < CvTerm > elements of the < CvMappingRule > are logically combined. The ‘requirementLevel’ attribute can have the values MAY, SHOULD or MUST depending on whether the association with the CV term is optional, recommended or mandatory. The attributes ‘useTerm’ and ‘allowChildren’ of the < CvTerm > element state, if the term itself or children of it can be used for data annotation at this place inside a data instance file. The attribute ‘isRepeatable’ states if the term can be repeated at this position or not and the Boolean value ‘useTermName’ specifies if the checking of the CV term is done on the ‘termName’ (if true) or on the termAccession (if false).

An example of such a < CvMappingRule > is given in the following, which states that in a mzIdentML file it is recommended that under the XPath “/MzIdentML/AuditCollection/Person/” there are < cvParam > elements describing the contact data of a person. The < cvParam > elements allowed here are all logical OR combinations of the three CV terms ‘contact address’, ‘contact URL’ and ‘contact email’:

< CvMappingRule id = “AuditCollectionPerson_rule”
  cvElementPath = “/MzIdentML/AuditCollection/person/cvParam/@accession” requirementLevel = “SHOULD”
scopePath = “/MzIdentML/AuditCollection/person” cvTermsCombinationLogic = “OR”>
  < CvTerm termAccession = “MS:1000587” useTermName = “false” useTerm = “true” termName = “contact address”
    isRepeatable = “true” allowChildren = “false” cvIdentifierRef = “MS” />
< CvTerm termAccession = “MS:1000588” useTermName = “false” useTerm = “true” termName = “contact URL”
  isRepeatable = “true” allowChildren = “false” cvIdentifierRef = “MS” />
< CvTerm termAccession = “MS:1000589” useTermName = “false” useTerm = “true” termName = “contact email”
  isRepeatable = “true” allowChildren = “false” cvIdentifierRef = “MS” />
</CvMappingRule >

In addition to the standard syntactic checks for well-formedness (i.e. if the XML file fulfills the XML syntax rules) and validity (i.e. if the XML file follows the structure defined in the corresponding XML schema), these mapping files thus allow an additional semantic checking of CV term usage in XML files [112–116]. In general, there might exist more than one mapping file per format, which could allow for different levels of stringency checking, e.g. checking MIAPE compliance (see next paragraph) or compliance to specific journal guidelines [15–18].

## 6 MIAPE compliance

To ensure that published experimental data fulfill basic requirements regarding reproducibility, transparency and secondary usage of the data, the MIBBI (Minimal Information for Biological and Biomedical Investigations) [110] project was founded. It describes minimal information checklists that data and metadata describing an experiment should fulfill. For proteomics, the MIAPE (Minimum Information about a Proteomics Experiment) [111] guidelines describe what information should be reported about an experiment, for example in a text document or a data file. A basic (text-based) mapping table defined together with each standard lists the possible locations of MIAPE requirements within the standard. Additional (computer-readable) mapping files and validators may be developed to allow checks for e.g. all steps between a “minimal sensible file” and a “strictly MIAPE-conform file”. A first implementation is [114]. Currently there are the following MIAPE guidelines defined: MIAPE-MS [117], MIAPE-MSI [118], MIAPE-GI [119], MIAPE-GE [120], MIAPE-CC [121], MIAPE-CE [122] and MIAPE-Quant [123]. The validators are either based on the PSI semantic validator framework [124], the underlying Java library used for developing the validators for the various HUPO XML-based proteomics standard formats, or are implemented locally or in web environments. The MIAPE compliance can also be tested by using the ProteoRed MIAPE web toolkit [125]. On the website [126] one can find links to the available validators for the various HUPO-PSI proteomics standards. All these validators check if the rules specified in the mapping file for the respective standards are fulfilled by a given instance data file.

## 7 Maintenance of the controlled vocabularies and ontologies

In the PSI community practice document [89] the HUPO-PSI working groups defined some guidelines for the development of controlled vocabularies. Since ongoing technological progress and the upcoming of new instruments and methods, an ontology is never complete, and steadily grows over time. Therefore the ontologies need a continuous maintenance. For the PSI-MS [70] ontology the maintenance procedure is as follows: Everyone in the proteomics community is free to subscribe to the psidev-ms-vocab mailing list [127] and to make proposals for new terms and/or improvements of the already existing psi-ms.obo ontology terms. After receiving a request for a new CV term the PSI ontology coordinator checks if the proposed term and its description, data type, parent terms and relations are sensible. It is also checked if the term is already part of other ontologies, e.g. MALDI imaging obo [65] or ChEBI [63] and if it is better to add them there or if the term isn't necessary because there exists already an attribute in the standard files, which describes the same fact. A term which passes all these checks is then included into the next release candidate of the obo file, which is sent to the three mailing lists psidev-ms-vocab@lists.sourceforge.net, psidev-pi-dev@lists.sourceforge.net and psidev-ms-vocab@lists.sourceforge.net for public discussion. If the proteomics community comes to consensus with the new term, then it is added to the next release version of the obo file, which is then made public at a CVS repository [128] and announced via the three mentioned mailing lists. A more detailed description of the PSI-MS maintenance process can be found at [Mayer et al., 2012, in submission].

## 8 Summary

In the last 10 years the proteomics community defined several modern standard formats (most of them XML-based) useful for the representation of the complex and large data sets faced in proteomics today. Because it is necessary to enrich these data with semantic information in order to annotate and make use of them effectively, the data standards refer to controlled vocabularies defined in ontology formats, of which the obo format is the one predominantly used today. In this manuscript, we briefly described the obo format and discussed some software tools for easily working with these files.

The integration of the terms defined in the ontologies into the XML data standards made it necessary to develop semantic validators for checking the correct use of the CV terms. For this, the validators make use of mapping files that complement the standard format defining XML schemas, and contain the rules for the correct usage of the CV terms. Also the conformance to the MIAPE and/or journal guidelines can be assured by additional mapping files governing the use of specific terms. Finally, the current procedure for maintaining the PSI mass spectrometry ontology psi-ms.obo was presented.

## Figures and Tables

**Table 1:** Important standard formats for use in proteomics.

Standard format	Use of CV/ontology	Website (accessed 11/2012)
JCAMP-DX [21]	None	http://www.jcamp-dx.org
ANDI-MS / netCDF [22]	None	http://enterprise.astm.org/filtrexx40.cgi?+REDLINE_PAGES/E1947.htm
mz5 / HDF5 [42,43]	Possible	http://software.steenlab.org/mz5
YAFMS [41]	PSI-MS	http://omics.pnl.gov/software/YAFMS.php
pepXML [56]	None	http://tools.proteomecenter.org/wiki/index.php?title=Formats:pepXML
protXML [57]	None	http://tools.proteomecenter.org/wiki/index.php?title=Formats:protXML
PSI-MI [37]	PSI-MI	http://www.psidev.info/mif
PSI-PAR [38]	PAR-CV	http://www.psidev.info/psi-par
mzML [24–26]	PSI-MS	http://www.psidev.info/mzml
TraML [31]	PSI-MS	http://www.psidev.info/traml
mzIdentML [27,28]	PSI-MS	http://www.psidev.info/mzidentml
mzQuantML [29,30]	PSI-MS	http://www.psidev.info/mzquantml
mzTab [45]	PSI-MS	https://code.google.com/p/mztab
imzML [35,36]	Imaging MS	http://www.maldi-msi.org
GelML [32]	sepCV	http://www.psidev.info/gelml
spML [33]	sepCV	http://www.psidev.info/search/node/spML

**Table 2:** Important ontologies, which are used in the proteomics field.

Ontology/CV	Prefix	Ontology file name	Website (accessed 11/2012)
Brenda tissue [62]	BTO	BrendaTissueOBO.obo	http://www.brenda-enzymes.info/ontology/tissue/tree/update/update_files/BrendaTissueOBO
Chemical entities of biological interest [63]	CHEBI	chebi.obo	http://obo.cvs.sourceforge.net/obo/obo/ontology/chemical/chebi.obo
Gene ontology [64]	GO	gene_ontology.obo	http://obo.cvs.sourceforge.net/obo/obo/ontology/genomic-proteomic/gene_ontology.obo
MALDI imaging ontology [65]	IMS	imagingMS.obo	http://www.maldi-msi.org/download/imzml/imagingMS.obo
PSI-Molecular Interactions [66–68]	MI	psi-mi.obo	http://obo.cvs.sourceforge.net/obo/obo/ontology/genomic-proteomic/protein/psi-mi.obo
PSI-Protein modifications [69]	MOD	PSI-MOD.obo	http://psidev.cvs.sourceforge.net/viewvc/psidev/psi/mod/data/PSI-MOD.obo
PSI-Mass Spectrometry [70]	MS	psi-ms.obo	http://psidev.cvs.sourceforge.net/viewvc/psidev/psi/psi-ms/mzML/controlledVocabulary/psi-ms.obo
Ontology for Biomedical Investigations [71]	OBI	obi.owl	http://www.obofoundry.org/cgi-bin/detail.cgi?id=obi
Phenotype Attribute Trait Ontology [72]	PATO	quality.obo	http://obo.cvs.sourceforge.net/obo/obo/ontology/phenotype/quality.obo
PRIDE [19] CV	PRIDE	pride_cv.obo	http://code.google.com/p/ebi-pride/source/browse/trunk/pride-core/schema/pride_cv.obo
Protein ontology [73]	PRO	pro.obo	http://obo.cvs.sourceforge.net/obo/obo/ontology/genomic-proteomic/pro.obo
OBO Relationship Ontology [74]	OBO_REL	relationship.obo	http://obo.cvs.sourceforge.net/obo/obo/ontology/OBO_REL/relationship.obo
PSI-Sample Processing and Separations [75]	SEP	sep.obo	https://psidev.svn.sourceforge.net/svnroot/psidev/psi/sepcv/trunk/sep.obo
Unimod modifications [76]	UNIMOD	unimod.obo	http://www.unimod.org/obo/unimod.obo
Units of measurement [77]	UO	unit.obo	http://obo.cvs.sourceforge.net/obo/obo/ontology/phenotype/unit.obo

**Table 3:** Software programs for accessing, browsing, creating, editing and manipulating ontology files.

Name	Category	Website (accessed 11/2012)
OBO-Edit [84]	Ontology editor	http://oboedit.org
Protégé [101]	Ontology editor	http://protege.stanford.edu
OLS (Ontology Lookup Service) [14]	Web service interface, Web portal	http://www.ebi.ac.uk/ontology-lookup
OLS dialog [103]	Java plug-in component	https://code.google.com/p/ols-dialog
OLSVis [104]	Visual browser	http://ols.wordvis.com
OBO-Explorer [105]	Ontology editor	http://www.aiai.ed.ac.uk/project/cobra-ct
NCBI BioPortal [13]	Web portal	http://bioportal.bioontology.org
